# Convallaria Majalis

**Published:** 1885-10-20

**Authors:** G. M. Garland

**Affiliations:** Assistant in Clinical Medicine, Harvard University, Boston, Mass.


### CONVALLARIA MAJALIS.

By G. M. Garland, M. D.,
Assistant in Clinical Medicine, Harvard University, Boston, Mass.

The following cases are offered as a contribution to the study of the virtues of convallaria majalis. They are too few to employ as a basis for general deductions, but they seem worthy of a record. The preparation used was the fluid extract of convallaria, which was made by Dr. E. R. Squibb, who sent it to me for trial.

### Case I.

James McK——, aged eighteen years, applied at the Boston Dispensary on October 16, 1883; patient had had rheumatism six times during the preceding nine years. He complained of pain in the cardiac region, and inability to lie on either side; was obliged to lie upon his back, and his nights were very restless; appetite good. There was dyspnœa on exertion. The heart's action was violent, and the carotids beat conspicuously. His head was shaken at each heart-beat. Radial pulse was 116 when patient was standing, and it almost disappeared on elevating the arm above the head. On auscultation the murmurs characteristic of aortic stenosis and aortic insufficiency were audible. There was excessive enlargement of the heart, so that the impulse was one inch outside of the left mammillary line. The urine was scanty; it contained albumen, blood, and bloody casts. There was some redness of the ankles.

Ordered ext. convallaria fl. m. x, four times daily.

October 19. Boy reports that he can sleep better; has less pain in the cardiac region; his heart does not beat so hard when he wakes up.

November 2. Patient knocked off work for two weeks, and has improved greatly. The urine has doubled in amount; has gained two pounds in weight; has now returned to work; sleeps well; can lie in any position.

Novemher 9. Patient left off his convallaria for a few days; his urine began to diminish in amount, and he did not feel so well; resumed the medicine, and began to improve again.

In this case, convallaria produced an immediate improvement in all the symptoms, although there were present albumen and casts in the urine. It has been stated that convallaria is useless under such conditions.

### Case II.

J. W. P——, forty years of age; saloon keeper.

October 1, 1883. Patient had rheumatic fever six years ago. Last June noticed shortness of breath and distress at the pit of the stomach, especially on walking; has not been a hard drinker; his complaints are numerous; has disagreeable beating in his neck on lying down, and is very short of breath—noticeable when he is walking; his heart beats irregularly, and when he wants to retire he is obliged to sit up in bed for a while before he can lie down; he feels a pressure in his face and neck, and suffers "fearful" with piles ; also, has nausea and retching every morning ; his abdomen has grown large and hard, and his legs are swollen ; belches gas frequently.

Examination of the man revealed the following conditions : Considerable jaundice of the skin and conjunctivæ ; ascitis, œdema of the lungs and legs, violent action of the carotids ; well marked Corrigan pulse. The cardiac dulness extended an inch to the left of the nipple ; loud murmurs were audible, but, owing to the irregular and violent action of the heart, they were hard to localize. Mitral and aortic regurgitations were considered probably present.

Ordered milk diet, and ext. conval. fl. m. x, four times daily.

October 5. Patient has kept his room since previous statement, but sits up every day ; feels less bloated ; breathes easier ; pulse 80–90 beats per minute, irregular and intermittent ; jaundice is diminishing.

October 10. Patient came to office after a trip down town ; reports great improvement ; can lie down and sleep comfortably ; circumference of his belly has diminished four inches, so that he can button his pantaloons now ; passes a large amount of urine—was up three or four times last night to urinate ; thinks he passed three quarts ; water is natural and light colored.

October 22. Reports favorable progress ; still has some beating in the stomach after eating ; pulse 88, thready but steady ; heart does not jump as it did, when patient lies down ; bowels have been costive, but the piles are all gone

November 2. Not so well ; piles again troublesome ; has to sit up in bed, before lying down ; terrible beating of the heart.

Ordered him to stop the convallaria, and take digitalis, m. x, three times daily.

November 4. Began with digitalis as directed. The first dose quieted his heart and stopped its throbbing ; last night, however, he began to have nausea, and this morning commenced to vomit—vomited what he had eaten, and then mucus at intervals. This afternoon find patient in bed ; heart's action is quiet in spite of the vomiting. This is the first time he has vomited since he began treatment with me.

Stopped the digitalis, and gave him quieting powders, for the stomach.

November 7. Patient continued to vomit until yesterday ; heart is quite steady, however ; pulse 60, slightly intermittent ; has lived on milk and Mellin's food ; urine contained no albumen, no blood, no casts.

Ordered digitalis, m. 2, three times daily.

November 10. Had a bad throbbing spell last night, but continued digitalis, and feels better today ; pulse, 48–51, sitting.

November 14. Had another vomiting spell, chiefly mucus—no food retained ; heart is quiet ; vomiting checked by morphine.

November 15. Pulse 36–40 ; vomiting stopped ; nose is very red. Related a curious freak of his eye-sight : said he had noticed, for a few days, when looking at white objects they appear yellow to him ; other objects look blue ; the silver armor of the soldiers at the theater looked gilded, and he was surprised when told that they were silvered. On white objects across the street, he sees one spot focussed yellow ; tops of horse's ears looks as if covered by a blue roof. Patient is prejudiced against the digitalis ; he thinks it disagrees with him, and he often feels worse soon after taking it ; he wished to return to the convallaria.

Accordingly, resumed that drug, m. x, three times daily.

November 18. Was seized last night with a terrible pain in the right side ; quieted it with morphine, and gave a tablespoonful of oil to move his bowels, which have been constipated.

December 14. Reports great improvement ; sleeps and eats well ; piles are gone again ; passed two to three quarts of water by night ; also, passes it freely by day, but less than during the night ; pulse 80, after walking.

February 9, 1884. Subsequent to the last entry, the patient moved away from Boston, and I never saw him again. He took a bottle of convallaria with him. He died upon the date of this entry. He was able to be about until one week before his death. His œdema returned, however, and he was obliged to take to his bed ; asked to be turned on to his side, and died as they turned him.

In this case, digitalis, evidently, distressed the patient, caused discomfort and vomiting. As soon as the digitalis was stopped and we returned to convallaria his stomach symptoms subsided, and he began to mend.—*New England Medical Monthly.*

